# Annotations

**Published:** 1904-10-08

**Authors:** 


### Annotations.

### The Futility of Lectures.

A distinguished professor of chemistry has recently expressed the opinion that the number of lectures to which students are compelled to listen is far in excess of what is either necessary or advisable. That is a proposition which has been announced on many occasions and somewhat curiously its sponsors have not infrequently been those engaged in the practice of teaching. It is not improbable, however, that it finds a response from a large proportion of the student world where facts as they are and not as they ought to be form the main theme for discussion. Considered from this aspect it needs no very extensive acquaintance with the organisation and practice of medical schools and other colleges to sustain the conviction that many, perhaps the majority, of lectures as actually delivered are absolutely useless. They neither succeed in conveying information to the student, nor enlarging his mental horizon, and, so far from arousing his enthusiasm for knowledge, they sadden his thoughts and waste his time. The explanation is that many lecturers fail to perceive that the purpose of the lecture has changed since the introduction of printed books. When knowledge was the possession of the few, and no method other than the spoken word existed for its distribution, students necessarily were compelled to wait on the accents of the master and to write down the words which fell from his lips. But with a cheap press such a performance is quite unnecessary. Yet many of our lecturers continue the primitive method and laboriously dictate from a manuscript facts which are equally well displayed in an ordinary text-book. That these uncorrected exercises in dictation—to call them lectures is to misname them—are useless, and worse than useless, will be allowed by everyone who has suffered from them. But the position is entirely different in regard to the teacher who is informed in his subject and has trained himself in his business—namely, in the art of clear and sympathetic exposition. His text is rather the relationships between facts than a mere statement of the facts themselves, and his object is to produce certain impressions on the minds and thoughts of his hearers and not only to add so many pages to their note-books. So conducted, lecturing is one of the most successful and widely appreciated methods of teaching, and those who cannot, or will not, learn its secret should be relegated to other spheres of activity. It would be a happy day for medical education could our colleges get rid of those "who cannot teach and will not learn."

### The Royal Commission on Sewage.

The Royal Commission on the Utilisation of Sewage has just put forth four folio volumes of a very elaborate report dealing with the application of crude or more or less prepared sewage and of trade refuse of various kinds to the land, and containing the histories of the working of several sewage-farms which have been kept under the careful observation of the Commissioners and of their professional advisers—chemical, bacteriological, and engineering. Perhaps the result that comes out most clearly is that sewage-farms may be conducted with moderate success under a great variety of conditions, but that they should be managed primarily in order to afford a satisfactory effluent, and only secondarily with reference to pecuniary return. Many contradictory opinions are quoted from accomplished expert and other witnesses, and the commissioners have not always accepted the responsibility of deciding between them. More than once, indeed, we have been reminded of the famous summing up by Mr. Justice Stareleigh in the historic case of Bardell *v.* Pickwick. "If Mrs. Bardell were right, it was perfectly clear that Mr. Pickwick was wrong, and if they thought the evidence of Mrs. Cluppins worthy of credence they would believe it, and, if they didn't, why they wouldn't." On the whole, we think it can hardly be said either that the results obtained have been equivalent to the time and labour spent in obtaining them, or that the evidence in support of sewage farming is sufficiently convincing to render the establishment of such farms at all an essential part of the duty of sanitary authorities.

### Throat Disease at Lincoln.

The recently issued report of the Medical Officer of the Local Government Board contains, among much other important matter, a detailed account of the two curious epidemics of obscure throat disease at Lincoln, which occurred in May 1902 and again in May 1903, both of them presenting many points of resemblance to scarlet fever, and both of them almost limited to the consumers of the same source of milk supply, which, however, was different in the two cases. An interesting history of the first of the epidemics was read before the Clinical Society by Dr. W. H. Brook, of Lincoln, in November 1902, and pointed out the differences between the symptoms and those of genuine scarlet fever, differences which were accentuated by the fact that the cases seemed to be scarcely, if at all, sources of infection to those who came in contact with them, as well as by the fact that in no instance could Klein's micrococcus scarlatinæ be discovered in the throat secretions. Dr. Klein did, however, succeed in isolating a pathogenetic yeast which produced disease and death in rodents, and all the circumstances seem to point to the conclusion that the cows may have obtained this yeast by contact with "rust" in the grass of a field in which they were pastured, and that it may have passed from their teats and udders into the milk. Some 200 cases of the disease appeared in the best residential part of Lincoln within a few days, and the epidemic, which proved fatal in three cases, then ceased as suddenly as it had commenced. There seems no doubt that the disease was one which had not previously been recognised or described, although, in previous instances of its occurrence, it may very possibly have been mistaken for scarlet fever. The full relation of the facts contained in an appendix to the report is from the pen of Dr. Darra Mair, by whom the occurrences were very completely inquired into, and it comprises an account of the bacteriological investigations of Drs. Klein and Mervyn Gordon. The history would repay careful study on the part of any practitioner who had an anomalous form of acute throat disease under his observation.

